# Editorial: Peripheral immune system and neurodegenerative disease

**DOI:** 10.3389/fnagi.2022.970042

**Published:** 2022-08-05

**Authors:** Ke Zhang, Chao Wang, Jun-Liang Yuan, Yu Deng

**Affiliations:** ^1^Key Laboratory of Cell Biology, Ministry of Public Health, Department of Developmental Cell Biology, China Medical University, Shenyang, China; ^2^Key Laboratory of Medical Cell Biology, Ministry of Education, China Medical University, Shenyang, China; ^3^Institute for Brain Science and Disease, Chongqing Medical University, Chongqing, China; ^4^NHC Key Laboratory of Mental Health (Peking University), Department of Neurology, National Clinical Research Center for Mental Disorders (Peking University Sixth Hospital), Peking University Sixth Hospital, Peking University Institute of Mental Health, Beijing, China; ^5^Department of Environmental Health, School of Public Health, China Medical University, Shenyang, China

**Keywords:** neurodegenerative diseases, Alzheimer's disease, Parkinson's disease (PD), amyotrophic lateral sclerosis (ALS), peripheral immune

Neurodegenerative diseases are a class of chronic and irreversible disorders characterized by progressive degeneration and loss of function of the central and/or peripheral nervous systems. The main pathological feature of neurodegenerative disease in the central nervous system (CNS) is selective neuronal loss in the brain and spinal cord, leading to cognitive and/or motor dysfunction. The immune system plays a variety of roles in the pathophysiology of neurodegenerative diseases. Current understanding of microglia from basic and clinical findings is as the main innate immune cells in the brain, which can be activated and involved in the neuroinflammation in nearly all neurodegenerative disorders. In recent years, many scientists have shifted their ground on conceptualizing neurodegenerative disease as a neuron-centric disease; rather, a close functional connection between the peripheral immune system and central nervous system has been increasingly acknowledged. An increasing number of circulating immune cells have been detected in neurodegenerative brains. In this regard, understanding how the peripheral immune system interacts with the central nervous system in terms of regulating the onset and development of neurodegenerative diseases assumes importance. Studies aim at exploring the role of the peripheral immune system in neurodegenerative diseases will help to identify new targets and improve the feasibility of therapeutic interventions. The manuscripts in this Research Topic focus on the relationship between the peripheral immune and neurodegenerative disease or neurodegenerative pathological changes. We highlight three specific themes in this topic: (1) peripheral innate/adapt immune and neurodegenerative disease; (2) immunology-related serum or plasma or blood platelet studies on neurodegenerative disease; (3) the crosstalk between the peripheral immunity and central nervous system.

The manuscripts in this Research Topic cover three main types of neurodegenerative disease: Alzheimer's disease (AD), Parkinson's disease (PD), and Amyotrophic Lateral Sclerosis (ALS). AD, as the most common type of dementia worldwide, has already affected over 50 million people. The lack of definite diagnostic biomarkers and effective treatments is the main cause of uncontrolled AD. The progression of AD is a dynamic process, from pre-symptomatic AD, mild cognitive impairment (MCI), to AD stages. Therefore, a cost-effective, easy-to-measure biomarker to identify subjects who will develop AD, especially at the pre-symptomatic stage is urgently needed. Qin et al. investigated serum biomarkers during different AD stages and potential novel protein biomarkers of presymptomatic AD. There are thirteen proteins in the serum that were significantly different in patients with AD or MCI group. Some proteins including cathepsin D, immunoglobulin E (IgE), epidermal growth factor receptor (EGFR), matrix metalloproteinase-9 (MMP-9), von Willebrand factor (vWF), haptoglobin, and phosphorylated Tau-181 (p-Tau181) correlated with all cognitive measures. They conclude the serum level of p-Tau181 might be broadly available to identify individuals with pre-clinical AD and assess the severity of AD. Huang et al. used a meta-analysis method systematically to evaluate the association of peripheral blood cell counts and indices with AD and MCI. The changes in leukocyte, lymphocyte, neutrophil, and CD8^+^ T cell counts, as well as the neutrophil-lymphocyte ratio and the CD4^+^/CD8^+^ ratio, are closely associated with AD, which provides us a potential diagnostic value clinical data. Besides peripheral functional immune blood cells, the complement system, an important arm of the innate immune system, is inextricably intertwined with the development of cognitive impairment. Li Z. et al. investigated and discussed the differences in complement activation pathways in cognitive impairment and type 2 diabetes mellitus (T2DM) with cognitive impairment, which provide scientific data on innate immune links between cognitive dysfunction and other diseases. In the treatment for AD, Yang et al. carried out a Randomized Controlled Trial to investigate the effects of sport stacking on the overall cognitive repairment and brain function recovery in patients with MCI and AD. It suggested that sport stacking may increase the level of neuroprotective growth factors and enhance neural plasticity. In addition, Peng and Wu reviewed a protein named Irisin, which is an exercise-stimulating cleaved product from transmembrane fibronectin type III domain-containing protein five in elderly dementia and cognitive impairment. One of the important roles of Irisin is that it can be regarded as a mediator of muscle brain cross talk to provide theoretical support for exercise therapy for patients with dementia. These findings suggested that both exercise and sport are beneficial to patients with elderly cognitive impairment, MCI, and AD.

Parkinson's Disease is the second most common neurodegenerative disease in the elderly with the fastest-growing morbidity. PD is mainly characterized by motor features, such as postural instability, bradykinesia, tremor, and rigidity, which are caused by selective loss of dopaminergic neurons in the substantia nigra pars compacta. The interaction between CNS-resident cells and peripheral immune cells in PD pathogenesis has attracted the attention of researchers. In this Research Topic, Zhang et al. systematically retrieved and evaluated the functions of natural killer cells (NK) in PD. NK cells maybe play a neuroprotective role in PD pathogenesis. Regulating the function of NK cells reveals novel targets for the management and treatment of PD. Li D. et al. comprehensively reviewed a famous factor PDGF (platelet-derived growth factors). The manuscript covers the classification, structure, biological functions, and pathogenic roles in PD of PDGF. In the course of PD, PDGF participated the pathogenesis through a variety of mechanisms such as regulating mitochondrial function, Ca^2+^ homeostasis, protein misfolding aggregation, and neuroinflammation. They also discuss the potential treatment strategy of PDGF as a target through multiple methods, especially in genetic treatment. In ALS, Yu et al. made a detailed review of crosstalk between the peripheral and central immune system from a neuroimmunological perspective which provides new insight into pathogenic mechanisms and innovative therapeutic approaches for ALS. The most noteworthy is Zang et al. comprehensively reviewed the crosstalk of central and peripheral immune systems in the three neurodegenerative diseases mentioned above. They conclude the role and molecular mechanism of the most main central immune cells (microglia and astrocytes) and peripheral immune cells (Monocytes, NK cells, T cells, Dendritic cells, and B cells) in these neurodegenerative disorders.

In summary, this Research Topic highlights the emerging role of the peripheral and central immune systems in common neurodegenerative diseases. These manuscripts provide us with the potential target in peripheral immune cells from the diagnosis to therapy. In the future, we certainly expect more studies to be added and the discussion to continue.

## Author contributions

KZ and CW decided the layout, wrote the manuscript, and acted as Editors for this Research Topic. All authors contributed to the article and approved the submitted version.

## Funding

This work was supported by the National Natural Science Foundation of China (81571046), a Key project of the Educational Department of Liaoning Province (LJKZ0755), and Shenyang Young and Middle-aged Innovative Talents Support Program (RC210240).

## Conflict of interest

The authors declare that the research was conducted in the absence of any commercial or financial relationships that could be construed as a potential conflict of interest.

## Publisher's note

All claims expressed in this article are solely those of the authors and do not necessarily represent those of their affiliated organizations, or those of the publisher, the editors and the reviewers. Any product that may be evaluated in this article, or claim that may be made by its manufacturer, is not guaranteed or endorsed by the publisher.

